# Electricity

**Published:** 1882-04

**Authors:** 


ELECTRICITY; ITS NATURE AND FORMS, with a Study on Electro-Physiology. By *C. W. Boyce, M. D.* Pp. 85. Chicago: W. A. Chatterton. 1880.

In these few pages, Dr. Boyce gives us a clear and interesting sketch of the different forms of electricity. Electricity plays a great part in disease and health. In its proper place it is an agent capable of doing good service in healing the sick. This little work tells its story in such clear and plain language, that none can misunderstand it.

